# Molecular Sieve-Modified Separator for High-Performance Lithium-Ion Batteries

**DOI:** 10.1186/s11671-020-03327-8

**Published:** 2020-05-13

**Authors:** Yuqiong Kang, Changjian Deng, Zhengyang Wang, Yuqing Chen, Xinyi Liu, Zheng Liang, Tao Li, Quan Hu, Yun Zhao

**Affiliations:** 1 Hoffmann Institute of Advanced Materials, Shenzhen Polytechnic, Shenzhen, 518055 China; 2 Division of Energy and Environment, Engineering Laboratory for the Next Generation Power and Energy Storage Batteries Graduate School at Shenzhen, Tsinghua University, Shenzhen, 518055 China; 3 Department of Chemistry and Biochemistry, Northern Illinois University, DeKalb, IL 60115 USA; 4 Department of Materials Science and Engineering, Stanford University, Stanford, CA 94305 USA; 5 Changsha Nanoapparatus Co., Ltd, Changsha, 410017 China

**Keywords:** Lithium-ion batteries, Separator, Molecular sieves, Water absorption, Electrospinning

## Abstract

Lithium-ion batteries (LIBs) are currently the most important energy storage system. Separators in the battery play a critical role in terms of the rate capability, cycle life, and safe operation. However, commercial separators exhibit poor electrolyte wettability and limited safety. It is also extremely important to eliminate the hazardous small molecules (e.g., H_2_O and HF) inside the battery to enhance the service life. Herein, a functionalized poly(vinylidene fluoride-co-hexafluoropropylene)@polyacrylonitrile (PVDF-HFP@PAN) separator modified by 4-Å molecular sieves (MS) was fabricated by hydrothermal method for LIBs. MS@PVDF-HFP@PAN separator exhibits high thermal stability and carbonate electrolyte wettability. In addition, it can lower the moisture value in the battery system to 13 ppm, which significantly improves the electrolyte quality. When the current density increased from 0.2 to 5 C, the discharging capacity of the cell with MS@PVDF-HFP@PAN declines from 177.6 to 143.2 mAh g^−1^, demonstrating an excellent capacity retention of 80.6%. The discharge capacity retention of NMC622 half-cell with MS@PVDF-HFP@PAN after 100 cycles is 98.6% of its initial discharge capacity, which is higher than that of a cell with the Celgard 2400 separator (91.9%).

## Background

Rechargeable lithium-ion batteries (LIBs) have become the dominant energy storage device for portable electronics due to high gravimetric energy and power density among commercial secondary batteries [1, 2]. However, much effort has been made to improve the service life of LIBs. The short service life of batteries results from inevitable side reactions during long-term cycling, which produces detrimental small molecules, such as H_2_O and HF [8, 9]. Particularly, there is an equilibrium reaction of the decomposition of LiPF_6_ to LiF and PF_5_ in electrolyte [3]. PF_5_ could react with trace amount of H_2_O in the electrolyte, producing HF and PF_3_O molecules. The formed HF could, in turn, enhance the production of H_2_O [2, 3]. As a result, the electrochemical performance and safety of LIBs could be severely impacted due to (1) the decomposition of cathode materials [4, 5], (2) the decomposition of solid electrolyte interface (SEI), (3) the decomposition of electrolyte [6, 7], and (4) significant self-discharging kinetics with trace H_2_O in batteries [8]. Therefore, the elimination of water molecules is very important for better LIBs.

Recently, many efforts have been devoted to improve the service life of LIBs by capturing HF or separating HF from electrode materials including surface coating of positive materials, adding inorganic/organic compounds for small molecular scavenging, functional electrolyte additives, etc. [9–14]. It is worth noting that the trace amount of water in the electrolyte could facilitate the formation of HF where H_2_O molecules provide hydrogen resources for the formation of HF [15]. Therefore, it is important to not only prevent the contact of HF and electrodes, but also eliminate the production of HF by the traceable water. Unfortunately, there is limited research on the removal of water in the electrolyte and the short service life of LIBs remains a challenge.

Herein, we provide a possible solution to capture water molecules by a unique separator. The separator is composed of poly(vinylidene fluoride-co-hexafluoropropylene)@polyacrylonitrile (PVDF-HFP@PAN) where homogeneous 4-Å molecular sieves (MS) are coated [11]. We characterize the proportion of MS, PVDF-HFP, PAN in MS@PVDF-HFP@PAN, the structure of MS, and the wettability of H_2_O and electrolyte. We also demonstrate the distribution and morphology of MS in PVDF-HFP@PAN under different conditions. Finally, the cycling performance of the as-obtained separators in NCM622 half-cell is presented.

## Presentation of the Hypothesis

Thermal runaway and water existence in the cell are detrimental to lithium-ion batteries. The introduction of functional separator made of modified molecular sieve contributes to improve the thermal stability and decrease the water content in the cell.

## Testing the Hypothesis

### Materials

PAN, PVDF-HFP (average Mw = 455,000, average Mn = 110,000, pellets), dimethylformamide (DMF, 99.8%), *N*-methyl-2-pyrrolidone (NMP, 99.5%), SiO_2_, Na_2_AlO_2_ and NaOH were purchased from Sigma-Aldrich. All of the reagents were used without further purification. Electrolyte (1 M LiPF_6_ dissolved in a mixture of ethylene carbonate (EC) and dimethyl carbonate (DMC) (v/v = 1:1), moisture about 50 ppm), poly(vinylidene fluoride) (PVDF, 99.5%), lithium metal foil (99.9%), copper foil (12 μm, 99.8%), aluminum foil (16 ± 2 μm, 99.54%), carbon black C45 and coin-type cell CR2032 were purchased from MTI Shenzhen Kejing Star Technology.

### Fabrication and Synthesis

The electrospun membrane was fabricated according to our previous work [16]. Briefly, the PVDF-HFP @ PAN membrane was prepared by dual-nozzle coaxial electrospinning. The core and shell solutions were prepared by 8 wt% PAN and 12 wt% PVDF-HFP dissolved in DMF. During the electrospinning process, the core and shell solutions were extruded at the rate of 0.54 mL h^–1^ and 1.08 mL h^–1^, respectively, with the electrospinning voltage at 15 kV. For the hydrothermal process, solution A and B were firstly prepared by dissolving the 3.6 g Na_2_AlO_2_ and 1.2 NaOH, 0.9 g Na_2_AlO_2_, 7.8 g NaOH, and 4.8 g SiO_2_ in 200 mL and 130 mL H_2_O, respectively. Then, solution A adds to solution B stirring for 2 days. After this, the hydrothermal growth of MS was carried out at 70 °C for 1 h in a sealed kettle by immersing the PVDF-HFP@PAN membrane in solution C.

### Materials Characterization

The thermal gravimetric analysis (TGA, STA 409 PC, Netzsch, US) measurement was carried out in airflow at a heating rate of 10 °C min^–1^ from room temperature to 900 °C. The morphology and elemental analysis of the membranes were characterized by scanning electron microscopy (SEM, SU-8010, Hitachi, Japan) and energy dispersive spectrometer (EDS, SU-8010, Hitachi, Japan). The X-ray diffraction (XRD, D8 Advance, Bruker, Germany ) with Cu Kα radiation was used to analyze the crystal structure of the MS over the range 10° ≤ 2θ ≤ 80° at the scan rate of 5° min^–1^. Contact angle (OCA15Pro, Dataphysics, Germany) was used to test the H_2_O wettability of the fabricated separator.

### Electrochemical Characterization

The electrochemical performance was measured using CR2032 coin-type half cells assembled in an argon-filled glove box with LiNi_0.6_Co_0.2_Mn_0.2_O_2_ (NCM) and lithium foil as the working electrode and the counter electrode, respectively. The NCM, carbon black C45 and PVDF with a mass ratio of 8:1:1 were dissolved in NMP to form a homogeneous slurry. The slurry was coated onto current collector via a doctor-blade coating method. The prepared electrode is dried in a vacuum oven at 110 °C for 12 h (mass loading about 4.2 mg cm^–2^). The galvanostatic discharge-charge cycling was performed in land system (CT2001A) over a range of applied voltage of 2.8–4.3 V at a constant C-rate of 0.1 C in the first cycle for activation and at 1 C in the following cycles.

## Implications of the Hypothesis

**Fig. 1:**
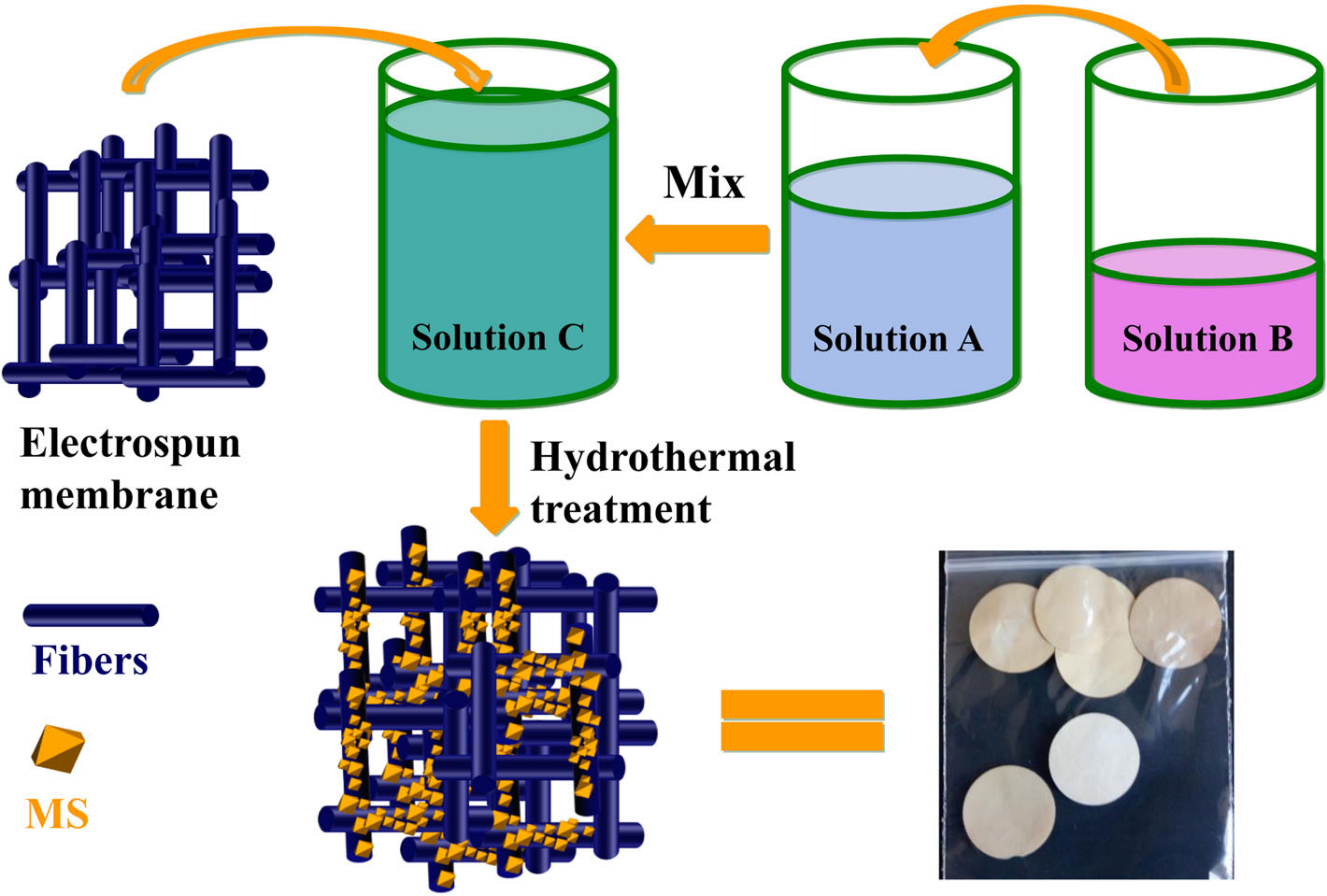
Schematic illustration of the fabrication of MS@PVDF-HFP@PAN. The obtained electrospun membrane immersing into the MS precursor solution can obtain the MS@PVDF-HFP@PAN by hydrothermal treatment

The process to fabricate the separator of MS@PVDF-HFP @ PAN is illustrated in Fig. 1: (1) PVDF-HFP@PAN separator with partially core-shell fibers is fabricated using coaxial electrospinning. (2) At the same time, the MS precursor solutions are prepared. (3) Then, MS@PVDF-HFP@PAN is obtained by putting the PVDF-HFP@PAN into MS precursor solutions for hydrothermal treatment. It is believed that crystals preferentially nucleate on the defects of materials. To expose partial PAN outsides of the fibers, which can be oxidized in the alkali condition for the growth of MS, high voltage is required during the process of electrospinning.

**Fig. 2:**
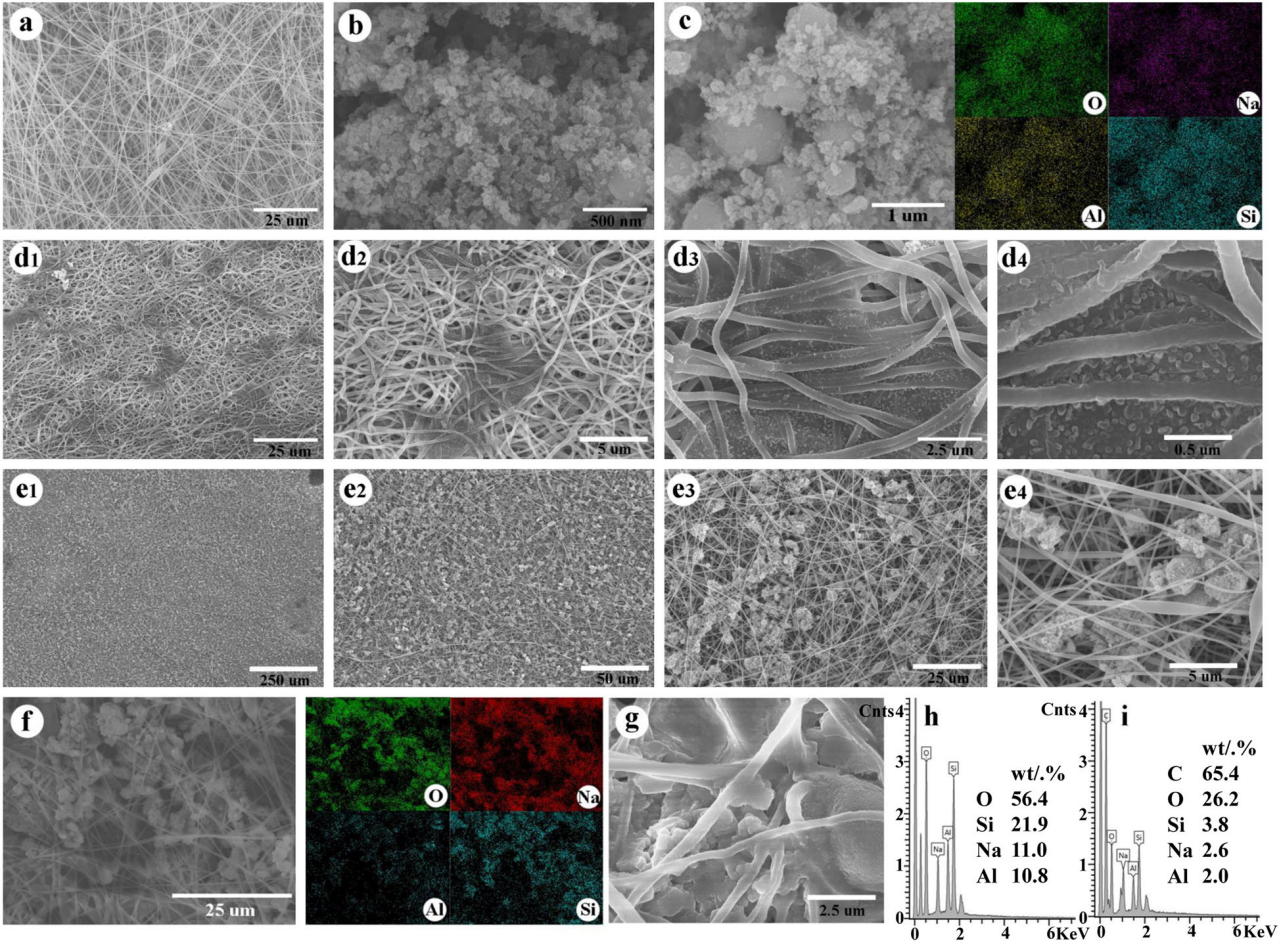
SEM images of **a** PVDF-HFP@PAN membrane, **b**, **c** MS particles, **d** PVDF-HFP@PAN after thermal treatment at 100 mL reactor, **e** PVDF-HFP@PAN after thermal treatment at 25 mL reactor, and **f**, **g** the enlarged MS@PVDF-HFP@PAN. **h**, **i** EDS results of MS and MS@PVDF-HFP@PAN, respectively

The morphology, elemental distribution of MS, PVDF-HFP@PAN, and MS@PVDF-HFP@PAN are examined by SEM as shown in Fig. 2. Although the diameters of MS range from ~ 100 to ~ 800 nm, the fibers of PVDF-HFP@PAN are quite smooth and homogeneous with little agglomerations (Fig. 2a). After the hydrothermal treatment of the PVDF-HFP@PAN membrane, a large number of particles is grown on the membrane structure (Fig, 2d1–4, e1–4). We investigated the hydrothermal condition to modify the distribution of nanoparticles. Two phenomena occur depending on the preparation conditions. It is found that the uniform nanoparticles are grown on the surface of fibers under high pressure. When PVDF-HFP@PAN is put in a 100 mL reactor with 20 mL solution C, the particles grow on the membrane in a special region with fine nanostructure (Fig. 2d1–4). Increasing the solution C to 70 mL in the reactor, the particles grow very uniformly in the membrane and the particle size is proper (Fig. 2e1–4). If there is no special mention, MS@PVDF-HFP@PAN prepared in a reactor with 70 mL solution C will be used as the separator for investigation. Since the ratio of O, Si, Na, and Al elements is similar in the materials of MS@PVDF-HFP@PAN and MS, to further verify that MS is grown on the PVDF-HFP@PAN, EDS is used. By comparing the mapping of the elements, it can be seen that some elements distributed in MS@PVDF-HFP@PAN are identical to the MS (Fig. 2b, c, f). The proportion of different elements is characterized by EDS (Fig. 2f) with the ratio of O:Si:Na:Al is 56:22:11:11 for MS@PVDF-HFP@PAN separator (Fig. 2i), which is consistent with that of in MS (Fig. 2h), proving that the MS has successfully grown on the PVDF-HFP@PAN film. It is worth noting that MS is grown onto the fibers tightly not just adsorb or restrict in the network of fibers (Fig. 2g).

**Fig. 3:**
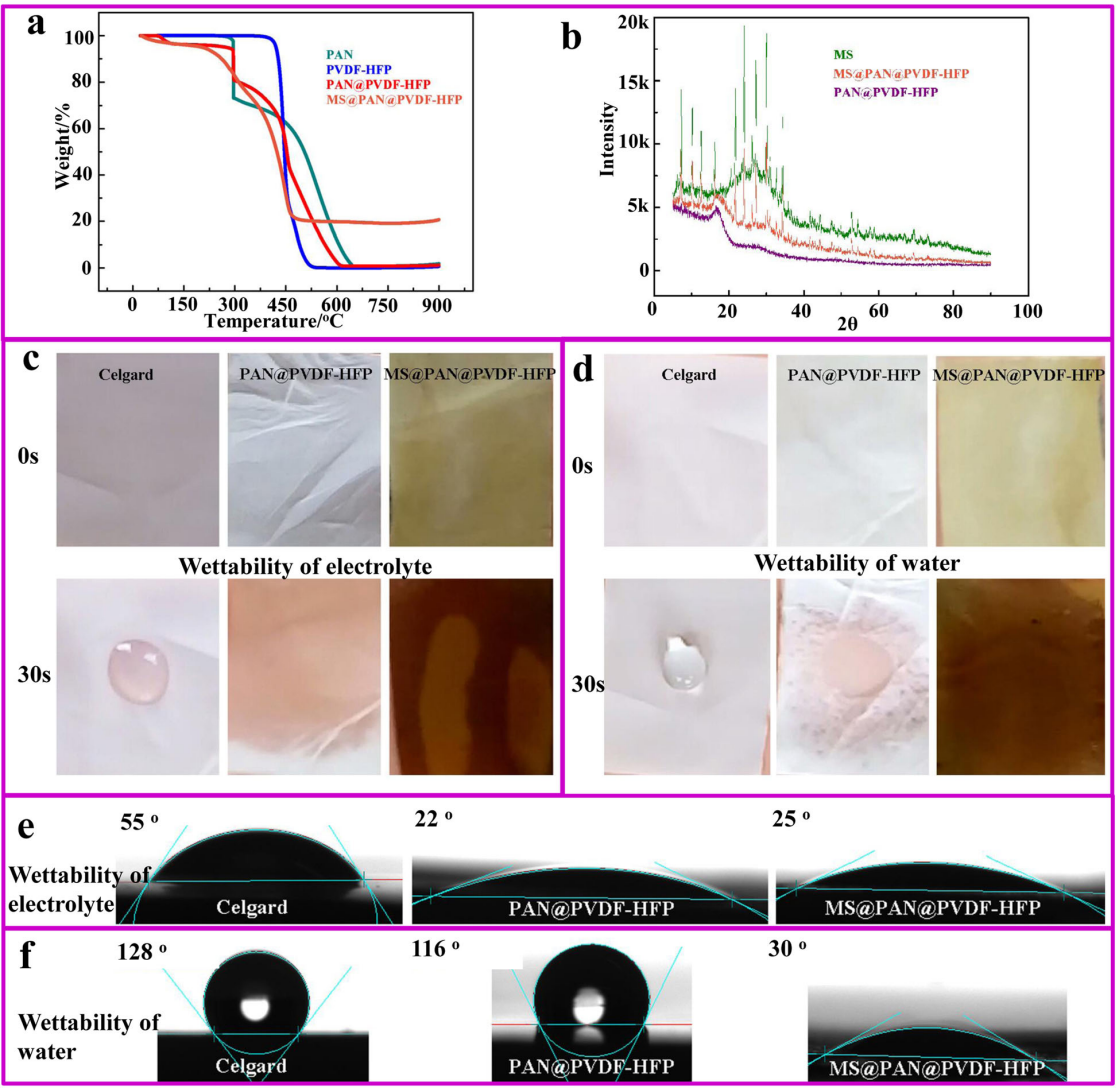
**a** XRD pattern of MS, PVDF-HFP@PAN, and MS@PVDF-HFP@PAN. **b** TGA curves of PAN, PVDF-HFP, PVDF-HFP@PAN, and MS@PVDF-HFP@PAN. **c**, **e** Electrolyte wettability of Celgard separator, PVDF-HFP@PAN, and MS@PVDF-HFP@PAN. **d**, **f** Water wettability of Celgard separator, PVDF-HFP@PAN, and MS@PVDF-HFP@PAN

With the MS functionalized PVDF-HFP@PAN separator in hand, the structure, and content of MS in the separator, and the wettability and contact angle of electrolyte and H_2_O for separator are characterized (Fig. 3). The crystal structure of MS, PVDF-HFP@PAN, and MS@PVDF-HFP@PAN was examined by XRD. As can be observed in Fig. 3a, PVDF-HFP@PAN shows broad hump rather than characteristic peaks, suggesting the amorphous feature. MS exhibits distinct diffraction peaks corresponding to type-A zeolite. MS@PVDF-HFP@PAN shows diffraction peaks corresponding to those of MS, suggesting that the crystalline structure of MS is maintained after the hydrothermal reaction and that MS is successfully embedded on the PVDF-HFP@PAN. The content of MS in the separator is determined by TGA. The PAN exhibits an obvious weight loss at 300 °C [17], then gradually decomposes at the temperature from 300 to 630 °C. The decomposition of PVDF-HFP occurs at 435 °C. When the PVDF-HFP and PAN are composited, PAN is first decomposed at 300 °C. The immediate weight drop at 435 °C is related to the removement of fluorine atom from PVDF-HFP. The gradual weight drop between 300 and 600 °C is due to PAN. In terms of MS@PVDF-HFP@PAN, the large weight drop at 300 °C disappears, suggesting the stage for PAN decomposition is changed due to the transformation of –CN in the process of hydrothermal treatment. There is 20 wt.% of MS when the temperature is 900 °C, suggesting the total content of MS in the separator is about 20 wt.%, demonstrating the successful fabrication of MS@PVDF-HFP@PAN.

Commercial separator is mainly fabricated by non-polar materials [17]. It has poor wettability for polar carbonate electrolytes, which limits the application in fast-charging batteries. The contact angle measurement is used to demonstrate the advantages of our materials in terms of electrolyte wettability and water absorption in Fig. 3c–f. The Celgard separator has a contact angle of 55° after with the liquid electrolyte on the surface for 3 s and it still remains the contact angle after the 30 s (Fig. 3c, e). However, the electrolyte droplet spreads out on the PVDF-HFP@PAN and MS@PVDF-HFP@PAN separator within 1 s, indicating MS has no obvious influence on the wettability of electrolyte. For the H_2_O wettability, Celgard separator is hydrophobic with a 128° contact angle for a long time (Fig. 3d, f). However, the H_2_O droplet is immediately adsorbed when contacting with MS@PVDF-HFP@PAN, showing great hydrophilic ability. The superior electrolyte and H_2_O wettability of the MS@PVDF-HFP@PAN separator are attributed to the micro-porosity of MS. According to the Karl Fischer Moisture Titrator, the MS@PVDF-HFP@PAN separator can lower the moisture value to ~ 13 ppm, which significantly improves the electrolyte quality [18]. Therefore, it can be expected that in addition to improving the rate performance and long cycle life of carbonate electrolyte-based batteries, the separator may show excellent performances in aqueous batteries as well.

**Fig. 4:**
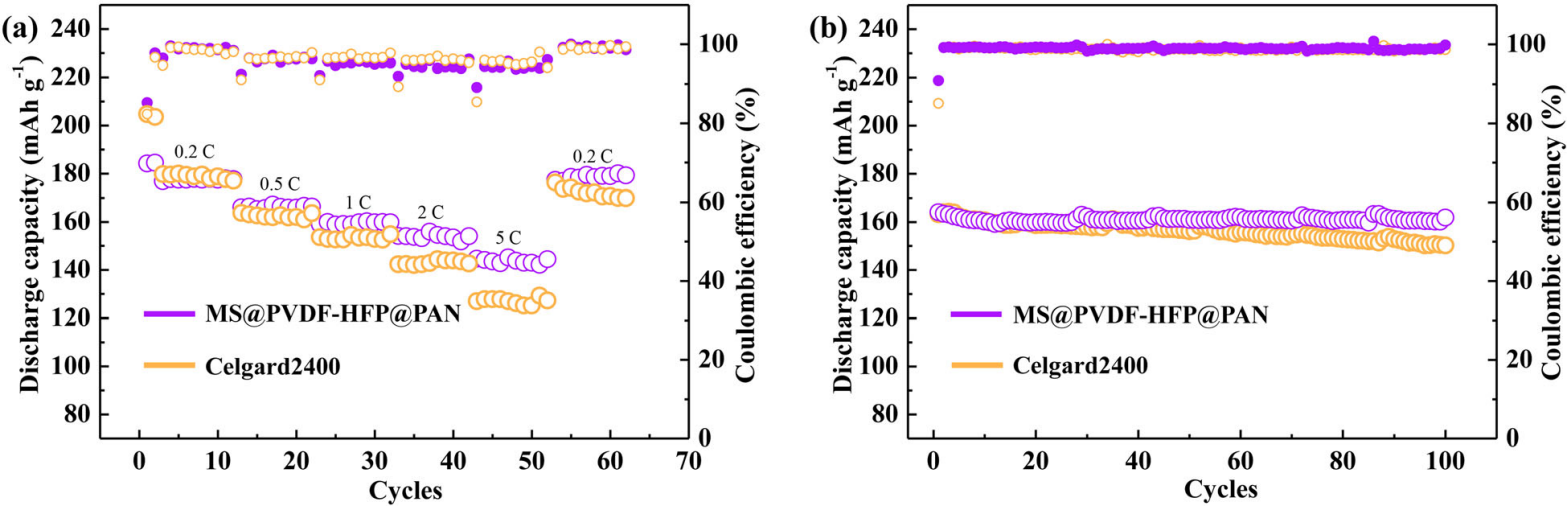
Electrochemical performances of the NMC622 half cells using MS@PVDF-HFP@PAN and Celgard separators. **a** The rate performances over a range of applied voltage of 2.8–4.3 V at a constant C-rate of 0.2 to 5 C. **b** Cycling performances under the same conditions

Figure 4a shows the rate capability of NMC622 half cells with either Celgard2400 or MS@PVDF-HFP@PAN as separators. When the current density increased from 0.2 to 5 C, the discharging capacity of the cell with MS@PVDF-HFP@PAN gradually declines from 177.6 to 143.2 mAh g^−1^, with a capacity retention of 80.6%. However, the capacity of the cell with Celgard2400 decreases sharply from 180.0 to 125.2 mAh g^−1^ with a capacity retention of 69.6%. It is required that the battery capacity lost is no more than 20% for fast charging in industry. Therefore, this unique separator offers a great opportunity for the development of fast-charge batteries.

The cycling stability of NMC622 half cells using the electrolyte with 50 ppm moisture at the current density of 1 C is tested to investigate the cycling performance of Celgard 2400 and MS@PVDF-HFP@PAN. As can be seen from Fig. 4b, the discharge capacity retention of the cell with MS@PVDF-HFP@PAN after 100 cycles was 98.6% of its initial discharge capacity, which is higher than that of a cell with the Celgard2400 as separator (91.9%). The improved performance may be attributed to the capture of moisture in the electrolyte by the MS on MS@PVDF-HFP@PAN. Therefore, the HF generation is suppressed which avoids the decomposition of cathode materials.

## Conclusions

We have prepared high wettability of carbonate electrolyte and water absorption separator by electrospinning technique and hydrothermal method. The MS in the separator can absorb trace water in the electrolyte suppressing the generation of HF, thus avoiding the collapse of the cathode materials by the acid attack, thereby improving the battery cycling stability. Combined with the high thermal shrink resistance of PVDF-HFP@PAN, MS@PVDF-HFP@PAN would play a more important role in the field of high-performance batteries. In addition, the material’s absorption for water makes it more suitable for aqueous batteries.

## Data Availability

All data are fully available without restriction.
